# 

**Published:** 1877-02

**Authors:** 


### BOOK NOTICES, REVIEWS, &c.

Some years ago, we had under our medical care a lady of great sweetness of character, the mother of a large family of children, to one of whom we sent a beautiful little book, beautiful in its mechanical execution, and as beautiful in sentiment, as it was true. Some days afterwards we inquired of the mother how her child liked the little book. "Really Doctor, I have not yet placed it in her hands, as I have not had an opportunity of reading it; for it is my habit, not to allow my younger children to read any book unless I have first examined it myself." This accomplished woman, with almost regal wealth at her command, had dismissed the world, with all the fascinations which a southern city can throw around social intercourse, and consecrated herself to the moral and religious tuition of her children. She soon passed away, but we feel quite sure, that she and the little ones she left behind her, will form a blest re-union above, a whole family saved in heaven, to be separated no more.

GLASSES.—In the September, 1876, number of the JOURNAL is an article entitled "Wearing Glassés," which has brought us many letters inquiring where the best glasses can be purchased. J. E. Spencer & Co., No. 16 Maiden Lane, New York, are, we believe, the largest manufacturers of spectacles and glasses in this country. They are perfectly reliable, and sell nothing but superior goods, at about the prices of the inferior and injurious glasses usually sold by peddlers. Send for descriptive pamphlet.

